# A Double Rate Localization Algorithm with One Anchor for Multi-Hop Underwater Acoustic Networks

**DOI:** 10.3390/s17050984

**Published:** 2017-04-28

**Authors:** Jingjie Gao, Xiaohong Shen, Ruiqin Zhao, Haodi Mei, Haiyan Wang

**Affiliations:** 1 Key Laboratory of Ocean Acoustics and Sensing, Northwestern Polytechnical University, Ministry of Industry and Information Technology, Xi’an 710072, China; gaojingj126163@126.com (J.G.); xhshen@nwpu.edu.cn (X.S.); rqinzhao@gmail.com (R.Z.); meihaodi@yeah.net (H.M.); 2 School of Marine Science and Technology, Northwestern Polytechnical University, Xi’an 710072, China

**Keywords:** localization, underwater acoustic networks (UANs), double rate, reference nodes

## Abstract

Localization is a basic issue for underwater acoustic networks (UANs). Currently, most localization algorithms only perform well in one-hop networks or need more anchors which are not suitable for the underwater environment. In this paper, we proposed a double rate localization algorithm with one anchor for multi-hop underwater acoustic networks (DRL). The algorithm firstly presents a double rate scheme which separates the localization procedure into two modes to increase the ranging accuracy in multi-hop UANs while maintaining the transmission rate. Then an optimal selection scheme of reference nodes was proposed to reduce the influence of references’ topology on localization performance. The proposed DRL algorithm can be used in the multi-hop UANs to increase the localization accuracy and reduce the usage of anchor nodes. The simulation and experimental results demonstrated that the proposed DRL algorithm has a better localization performance than the previous algorithms in many aspects such as accuracy and communication cost, and is more suitable to the underwater environment.

## 1. Introduction

Underwater Acoustic Networks (UANs) are deployed in the ocean and equipped with a lot of low-cost nodes which are able to communicate with each other via wireless acoustic signals. Over the last few years, more and more researchers have had growing interest in UANs, because UANs can be used in target detection or monitoring. Compared with traditional array sensors, UANs can improve the detection accuracy and monitoring scope and fulfill the needs of a multitude of underwater applications such as oceanographic data collection, ecological applications and military underwater surveillance, etc. [1].

There are two kinds of UANs in the underwater environment: the static networks and the mobile networks. (1) The static network is composed of nodes sinking to the bottom of sea. This kind of network has a fix topology and it is easier for nodes to communicate with each other; (2) One of the mobile networks is composed of nodes which float in the sea and drift with the tide and wave. The other one has some mobile nodes such as AUV (Autonomous Underwater Vehicle) which can travel through the region, increase the monitoring scope, but increase the cost as well. The topology of the mobile network is variational and the communication is effected by the mobility. In this paper, we mainly focus on the static underwater networks.

Localization is a major issue for UANs. It contains source localization and self localization [2]. In this paper, we focus on the research of the self localization algorithm. Self localization is the procedure that makes all the unknown nodes in UANs get their positions. All the routing protocols or detection applications should be on the basis of the self localization information. Therefore, it is important that research on the self localization algorithms for UANs takes place in order to make it perform better. Different from terrestrial networks, the localization algorithm for UANs suffers from some problems because of the characteristics of underwater environment: (1) Anchors are nodes which know their locations from GPS or manual measurement and help other unknown nodes to obtain their positions more accurately. However, GPS cannot be directly used in an underwater environment because of the serious attenuation. Hence, using a few anchors to get high localization performances is one of the key problems that should be solved in UANs; (2) Underwater acoustic channels are characterized by long propagation delays and limited bandwidth. These characteristics introduce challenges in terms of localization schemes design; (3) Different from terrestrial networks, UANs are always sparse and have limited power because of the nodes’ cost and battery capability [3,4,5].

Recently, some localization algorithms have been proposed such as UPS (Underwater Positioning System), WPS (Wide Coverage Positioning System), MSL (Multi-Stage Localization) algorithm, DV-hop (Distance Vector-Hop) algorithm, MDS-MAP (Multidimensional Scaling-MAP), IMDS (Improved MDS) and AAL (AUV-Aided Localization) algorithm, etc. UPS can only be used in one-hop UANs which reduces the network scale. MSL, DV-hop, MDS-MAP, IMDS and AAL can be used in multi-hop networks.However, MSL will have a large error accumulation due to the stages’ division; AAL uses an AUV to travel through the network which increases the cost of localization process; DV-hop and MDS-MAP use the range or communication connectivity information to estimate the position of each node in multi-hop networks which have a better performance in regular networks and more anchors; IMDS improved MDS by distance matrix refinement, but also improves computational complexity, which is not easy to accomplish for an underwater system [6,7,8,9,10,11,12,13,14,15,16].

In this paper, we proposed a Double Rate Self Localization Algorithm with One Anchor for Multi-hop Underwater Acoustic Networks (DRL). Firstly, a Double Rate Scheme is proposed. The scheme separates the localization process into two modes, low-rate and high-rate transmission mode, by choosing appropriate bit duration which can both increase the range measurement accuracy in multi-hop networks and ensure the transmission rate in UANs. Secondly, we present an Optimal References Selection Scheme that only needs one anchor and can reduce the influence of references’ topology on localization accuracy. Above all, the proposed DRL algorithm can be used in multi-hop UANs, increase the localization accuracy and only needs one anchor to accomplish the localization procedure which is more suitable for the underwater environment.

The paper is organized as follows: Section 2 describes the structure of UANs in this paper; then the double rate self localization algorithm with one anchor is proposed in Section 3; Section 4 analyses the simulation results of the proposed algorithm; the real experiment results are analyzed in Section 5; finally, Section 6 draws a conclusion.

## 2. The Network Architecture

**Figure 1:**
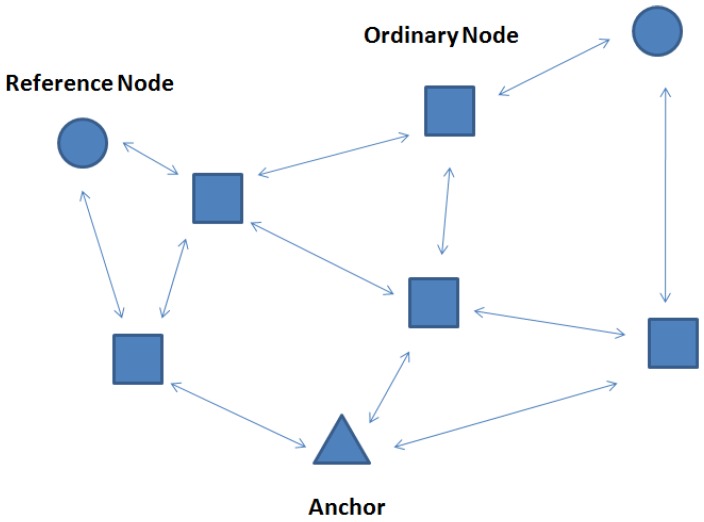
The Network Architecture.

In this paper, the proposed algorithm is used in a statistic network. Nodes in the network are all motionless. By using the proposed algorithm, we can accomplish the localization task by using only one anchor. The network architecture is shown in Figure 1.

The UANs are comprised of anchor and unknown nodes. Unknown nodes consist of reference nodes and ordinary nodes. The specific descriptions are as follows:

(1) Anchor: A node that knows its accurate position previously by GPS or manual arrangement. Other nodes can be localized by directly or indirectly communicating with it. In this paper, we only need one anchor to accomplish the localization procedure.
(2) Reference Nodes: Reference nodes are selected by the anchor to help it accomplish the localization procedure and can get the positions from the anchor. In this paper, we proposed an Optimal Selection Scheme of reference nodes to increase the localization accuracy.
(3) Ordinary Nodes: Ordinary nodes are sensor nodes which only receive the signals form anchors and references to get the positions.

In the proposed DRL localization algorithm, firstly, the reference nodes are selected from the anchor and their positions are obtained. Then, other ordinary nodes receive the signals from both the anchor and the newly selected reference nodes to accomplish the localization procedure.

## 3. The DRL Algorithm

DRL is a range-based localization algorithm. It can be used in static or relatively static UANs. The proposed DRL algorithm includes two schemes. (1) a Double Rate Scheme is proposed for multi-hop UANs which separates the localization process into two procedures by changing the bit duration $T_{s}$. Each procedure uses different transmission modes including the high rate transmission mode (HM) and the low rate transmission mode (LM) to make the range measurement in the multi-hop UANs more accurate while maintaining the data transmission rate; (2) An optimal selection scheme of reference nodes is proposed to accomplish the localization procedure with only one anchor and reduce the influence of anchor and references’ topology on the localization performance to increase the localization accuracy as well.

### 3.1. The Double Rate Scheme

Recently, most of the localization algorithms have a good performance only for one-hop UANs. However, one-hop UANs have a limitation on the detection range and other networks’ applications. In this paper, we mainly focus on the multi-hop networks which can increase the network’s scale, but introduce a challenge in terms of ranging accuracy which will ultimately have an effect on the localization performance.

In this section, we propose a Double Rate Scheme by analyzing the relationship between bit duration and transmission range in an underwater environment. In this scheme, we separate the localization process into two procedures and each procedure uses a different transmission mode to increase the ranging accuracy in multi-hop UANs while maintaining the data transmission rate.

#### 3.1.1. The Relationship between Code-Width and Transmission Range

The sonar equation is shown in Equation (1).

(1) SL-TL-NL=SNR

SL is the Source Level which is connected with the nodes’ property;

TL is the Transmission Loss;

NL is the Noise Level;

TL and NL should be selected according to the environment;

SNR is the Signal to Noise Ratio;

All of the parameters above are measured by dB.

(2) $$SNR=10\mathrm{lg}\frac{P_{s}}{P_{n}}=10\mathrm{lg}\frac{P_{s}}{N_{0}B}$$

$P_{s}$ is the signal power;

$P_{n}$ is the noise power;

$N_{0}$ is the unilateral power spectral density of Gaussian white noise;

*B* is the bandwidth of signal; for binary data,

(3) $$B=\frac{2}{T_{s}}$$

$T_{s}$ is the bit duration of signal. $T_{s}$ means the transmission time duration of one bit data.

From Equations (1)–(3), we can see that if $T_{s}$ of transmitted signal is increased *N* times, the bandwidth *B* will reduce to $\frac{1}{N}B$ and SNR will be increased by 10lgNdB. While, if we keep SL, $N_{0}$ and SNR be unchangeable, the TL will be ultimately increased by 10lgNdB.

TL=20lgr+αr

α is the is the coefficient of sound absorption. $\alpha =0.036f^{\frac{3}{2}}dB/km$. *f* is the frequency of transmission signal. *r* is the transmission distance.

(4) $$\begin{array}{cc} TL^{\prime } & =TL+10\mathrm{lg}N\\ & =20\mathrm{lg}r+\alpha r+10\mathrm{lg}N\\ & =20\mathrm{lg}r^{\prime }+\alpha r^{\prime } \end{array}$$

From Equation (4) we can see that the increase of bit duration $T_{s}$ will finally enlarge the transmission distance *r* which will make the ranging signal cover a larger scope and improve the distance measurement accuracy in multi-hop networks.

#### 3.1.2. The Double Rate Scheme in DRL Algorithm

Based on the relationship between $T_{s}$ and the transmission range, in this paper, we propose a Double Rate Scheme that separates the localization process into ranging procedure and localization procedure. Each of them uses different transmission mode by choosing proper $T_{s}$.

The two transmission modes are defined as low-rate transmission mode (LM) and high-rate transmission mode (HM), which are used in the ranging and localization procedure respectively.

(1) Low-rate Mode(LM): The signal of the low-rate mode has a large code-width which could transmit a great distance based on Equation (3). Hence, the LM signal can be used in the ranging procedure by choosing an appropriate code-width so that the signal could transmit across the network to increase the ranging accuracy in multi-hop networks.
(2) High-rate Mode(HM): In contrast, the signal of the high-rate mode has a small code-width which could only transmit a one hop distance. However, signal of HM could be used in the localization procedure to transmit a mass of data at a high rate.

According to the analysis above, the proposed Double Rate Scheme could improve the ranging accuracy in multi-hop networks as well as maintaining the data transmission rate.

### 3.2. The Optimal Selection Scheme of Reference Nodes for One Anchor UANs

The proposed DRL algorithm only needs one anchor to accomplish the localization procedure with high accuracy. The only anchor selects two unknown nodes in UANs as references to help. However, different references’ selection has different localization performance. Hence, in this section, we propose an Optimal Selection Scheme of reference nodes to reduce the influence of references’ topology on localization accuracy.

Assume that P sensor nodes are randomly distributed in a $L1\times L_{2}$ region including one anchor $N_{0}\left(x_{N0},y_{N0}\right)$ and P-1 unknown nodes. Two reference nodes $N_{1}\left(x_{N1},y_{N1}\right)$ and $N_{2}\left(x_{N2},y_{N2}\right)$ are selected by $N_{0}$ from all unknown nodes and the rest of them are ordinary nodes $X_{i}\left(x_{i},y_{i}\right)$, 1≤i≤M, M=P-3. The distance between $i^{th}$ ordinary node and $N_{0}$, $N_{1}$, $N_{2}$ are expressed by $d_{i0}$, $d_{i1}$ and $d_{i2}$ respectively.

(5) $$d_{ij}=∥X_{i}-N_{j}∥+n_{ij}\;\left(i=1,2,\cdots ,M;j=0,1,2\right)$$

$n_{ij}$ is the additive measurement noise error.

The least square (LS) estimation of Equation (5) is

(6) $$\underset{X_{i}}{\arg\min}\;\;\sum_{i=1}^{M}\sum_{j=0}^{2}(d_{ij}-∥X_{i}-N_{j}{∥)}^{2}.$$

The resulting estimation function (6) is a non-linear form. By expensing and subtracting the Equation (6), we get

(7) $$\begin{array}{c} \left\{\begin{array}{c} x_{N0}^{2}-x_{N2}^{2}-2\left(x_{N0}-x_{N2}\right)x_{i}+y_{N0}^{2}-y_{N2}^{2}-2\left(y_{N0}-y_{N2}\right)y_{i}=d_{i0}^{2}-d_{i2}^{2}\\ x_{N1}^{2}-x_{N2}^{2}-2\left(x_{N1}-x_{N2}\right)x_{i}+y_{N1}^{2}-y_{N2}^{2}-2\left(y_{N1}-y_{N2}\right)y_{i}=d_{i1}^{2}-d_{i2}^{2} \end{array}\right. \end{array}$$

Then we linearize (6) into Equation (7) and derive the solution as in Equation (8).

(8) $$X_{i}={\left(A^{T}A\right)}^{-1}A^{T}b_{i},1\leq i\leq M$$

$$A=\left[\begin{array}{cc} 2(x_{N0}-x_{N2}) & 2(y_{N0}-y_{N2})\\ 2(x_{N1}-x_{N2}) & 2(y_{N1}-y_{N2}) \end{array}\right]$$

$$b_{i}=\left[\begin{array}{c} x_{N0}^{2}-x_{N2}^{2}+y_{N0}^{2}-y_{N2}^{2}+d_{i2}^{2}-d_{i0}^{2}\\ x_{N1}^{2}-x_{N2}^{2}+y_{N1}^{2}-y_{N2}^{2}+d_{i2}^{2}-d_{i1}^{2} \end{array}\right]$$

$$X_{i}=\left[\begin{array}{c} x_{i}\\ y_{i} \end{array}\right]$$

According to the analysis above, we can also get the estimated location error of node *i* $E_{i}$.

(9) $$\begin{array}{cc} E_{i} & ={\left(b_{i}-\mathrm{AX}\right)}^{T}\left(b_{i}-\mathrm{AX}\right)\\ & ={\left(b_{i}-A{\left(A^{T}A\right)}^{-1}A^{T}b_{i}\right)}^{T}\left(b_{i}-A{\left(A^{T}A\right)}^{-1}A^{T}b_{i}\right)\\ & =b_{i}^{T}\left(I-A{\left(A^{T}A\right)}^{-1}A^{T}{b|}_{i}\right) \end{array}$$

Hence, the estimated location error *E* for the whole network with *M* nodes is shown as Equation (9).

(10) $$E=\sum_{i=1}^{M}E_{i}=\sum_{i=1}^{M}b_{i}^{T}\left(I-A{\left(A^{T}A\right)}^{-1}A^{T}b_{i}\right)$$

Then we can find the optimum reference nodes $\left(x_{N1},y_{N1}\right)$,$\left(x_{N2},y_{N2}\right)$ from *E* to get the minimum location error. Transform Equation (8) into an optimization problem.

(11) $$\begin{array}{c} \underset{\left(x_{N1},y_{N1}\right),\left(x_{N2},y_{N2}\right)}{\arg\min}\;\;\sum_{i=1}^{M}b_{i}^{T}\left(I-A{\left(A^{T}A\right)}^{-1}A^{T}b_{i}\right)\\ s.t.\;\left\{\begin{array}{c} A=\left[\begin{array}{cc} 2(x_{N0}-x_{N2}) & 2(y_{N0}-y_{N2})\\ 2(x_{N1}-x_{N2}) & 2(y_{N1}-y_{N2}) \end{array}\right]\\ b_{i}=\left[\begin{array}{c} x_{N0}^{2}-x_{N2}^{2}+y_{N0}^{2}-y_{N2}^{2}+d_{i2}^{2}-d_{i0}^{2}\\ x_{N1}^{2}-x_{N2}^{2}+y_{N1}^{2}-y_{N2}^{2}+d_{i2}^{2}-d_{i1}^{2} \end{array}\right]\\ 0\leq x_{N1}\leq L_{1},0\leq x_{N2}\leq L_{1}\\ 0\leq y_{N1}\leq L_{2},0\leq y_{N2}\leq L_{2} \end{array}\right. \end{array}$$

$N_{0}\left(x_{N0},y_{N0}\right)$, $N_{1}\left(x_{N1},y_{N1}\right)$ and $N_{2}\left(x_{N2},y_{N2}\right)$ should not be in a line. Thus, the proposed algorithm should not work in a linear network structure.

The optimization problem (11) can be solved using interior point methods. According to the objective function, we find the optimal references selection $N_{1}\left(x_{N1},y_{N1}\right)$ and $N_{2}\left(x_{N2},y_{N2}\right)$ in the constraint area that makes the localization error to be minimum.

DRL can be extended to the 3-dimensional networks by selecting three reference nodes.

### 3.3. The Implementation of DRL Algorithm

We also assume that the underwater network contains one anchor $N_{0}$, 2 reference nodes $N_{1}$ and $N_{2}$ selected by the anchor, M ordinary nodes. Set $N_{0}$ be the origin of coordinate, $N_{0}\left(0,0\right)$.

Based on the scope of underwater network and nodes’ power, we choose an appropriate code-width $T_{s}$ for the Double Rate Scheme.

The network uses TDMA (Time Division Multiple Address) protocol and TOA (Time of Arrival) ranging method [17,18] to reduce the transmission interference in UANs.

Step 1: The anchor $N_{0}$ broadcasts “hello” to the other nodes with the sending time stamp by LM mode.

Step 2: Same as Step 1, other nodes broadcast time stamp information by LM mode to the whole network.

Step 3: Every node can make some distance between itself and the other nodes, then send the ranging information to the anchor by HM mode.

**Figure 2:**
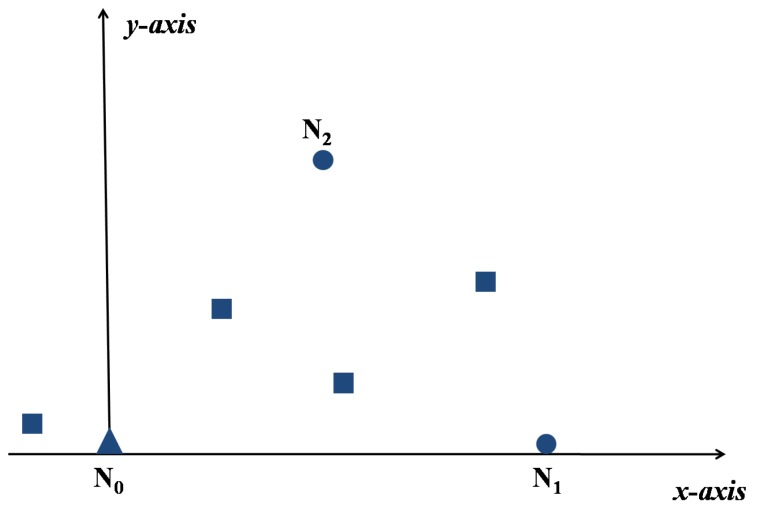
The relative coordinate system.

Step 4: The anchor $N_{0}$ selects the optimal reference nodes $N_{1}$ and $N_{2}$ according to Equation (10) with the ranging information in Step 3. Then by setting $N_{0}N_{1}$ be the *x*-axis, $N_{2}$ in the first quadrant, we can get the relative coordinate system as shown in Figure 2.

Step 5: $N_{0}$ gets the location information of each node and the topology of whole network according to Equation (8). Then the localization procedure ends.

**Figure 3:**
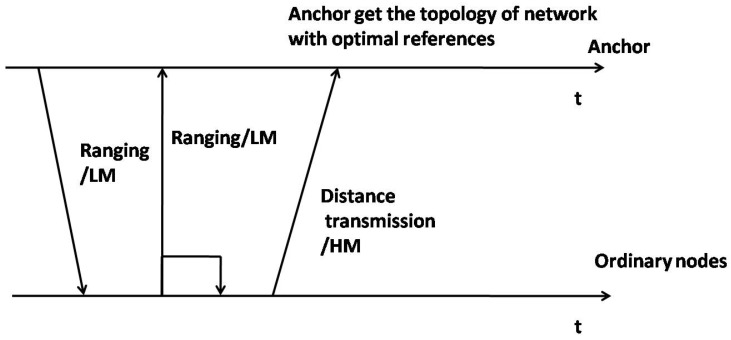
The localization process.

The localization process is shown in Figure 3.

If the scope of UANs is out of the range of the LM transmission mode, the localization procedure should be accomplished by different clusters which are divided by the transmission range of LM mode. The boundary nodes which have obtained their positions will be the anchor in new cluster and the localization procedure will end when all the unknown nodes have been localized.

## 4. Performance Analysis and Simulation Results

In this section, the proposed DRL algorithm is simulated and compared to some classic algorithms to evaluate its performance.

MSL, MDS-MAP, DV-hop are selected as the reference methods since all of them can be used in multi-hop networks and have low computation complexity which are more suitable for an underwater environment. Besides, the MSL algorithm only needs a few anchors to accomplish the localization procedure for multi-hop networks which is often used in UANs.

Twenty nodes are deployed in a 1000 × 1000 2-dimensional area and one of them is the anchor.

### 4.1. The Relationship between Code-Width and Transmission Range

According to the nodes’ property and the environment characteristic, we set SL=134dB, NL=70dB, SNR=4dB, the carrier frequency fs=10KHz.

If we increase the bit duration $T_{s}$ from 1ms to 25ms, the transmission range changes as in Figure 3.

**Figure 4:**
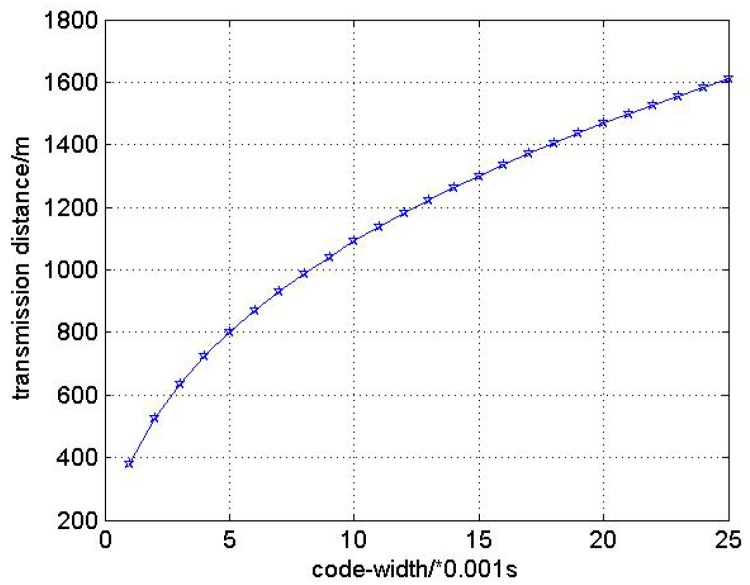
The relationship between code-width and transmission range.

In Figure 4 we can see that the transmission range increases along with the increase of code-width. When the code-width equals 1 ms, the transmission range is about 400 m. While the code-width changes to 11 ms, the transmission range increases to about 1100 m. Hence, we can choose the proper code-width of signal from the line in Figure 3 for HM and LM transmission modes receptively to make the distance measurement of multi-hop networks more accurate.

### 4.2. The Localization Performance

In this section, we evaluate the localization performance of DRL algorithm from four aspects: the effect of range error; the relationship between nodes’ number and localization error; the influence of reference nodes’ topology; and the communication cost.

Nodes in UANs are deployed in a 1000 × 1000 2-dimensional area randomly. Set $T_{s}$ be 1 ms and 11 ms for LM and HM modes receptively. We used 1000 Monte Carlo experiments with different network topologies in each test to average the effectiveness of topology variation.

#### 4.2.1. Test1: the Effect of Range Error

**Figure 5:**
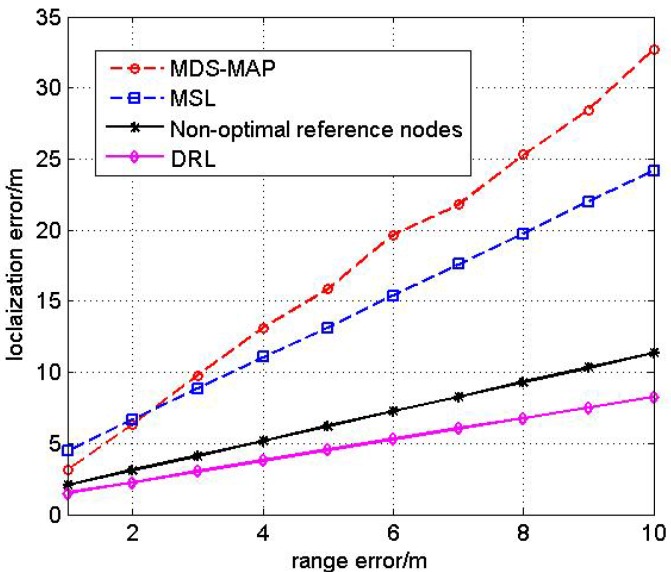
Comparison of different localization algorithms with range error.

According to Equation (5), we assume that the range error is white Gaussian noise with the variance $\sigma^{2}$ increasing from 1 m to 5 m. Compare the RMSE (Root Mean Square Error) of four different algorithms and the result is shown in Figure 5.

From the simulation result, we can see that MDS-MAP and MSL algorithms have a large localization error and the localization performance of the new proposed DRL algorithm is better than the other three approaches. The simulation result also demonstrates that the proposed Double Rate Scheme can increase the ranging accuracy in multi-hop networks which will improve the localization performance as well. The proposed optimal selection scheme of reference nodes can increase the accuracy further which means that DRL has the least localization error of the four algorithms.

#### 4.2.2. Test2: The Relationship between Nodes’ Number and Localization Error

**Figure 6:**
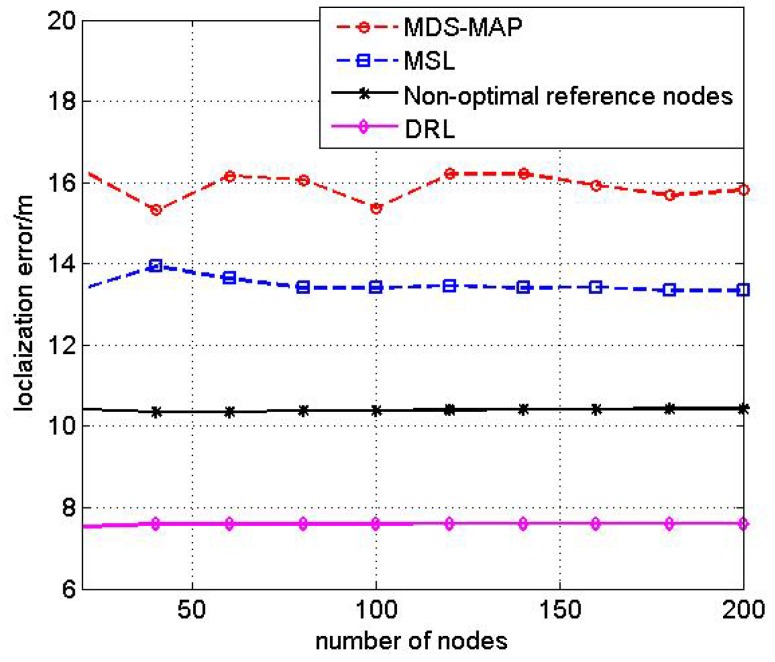
Comparison of different algorithms with number of nodes.

In this test, we analyze the impact of nodes’ number on the localization performance. Fix the Gaussian noise variance $\sigma^{2}$ to be 5 m and increase the number of nodes from 20 to 200. Figure 6 shows the impact of nodes’ number on localization error of DRL, MDS-MAP, MSL and the Double Rate Scheme with no reference nodes.

From the simulation result, we can see that the proposed DRL algorithm and the Double Rate Scheme with no reference nodes algorithm are less sensitive to the nodes’ number. MSL and MDS-MAP are affected by the number of nodes because the increase of nodes’ number will improve both the communication connectivity and enlarge the error accumulation. The localization results for MDS-MAP and MSL are irregular.

#### 4.2.3. Test3: The Influence of Reference Nodes’ Topology

**Figure 7:**
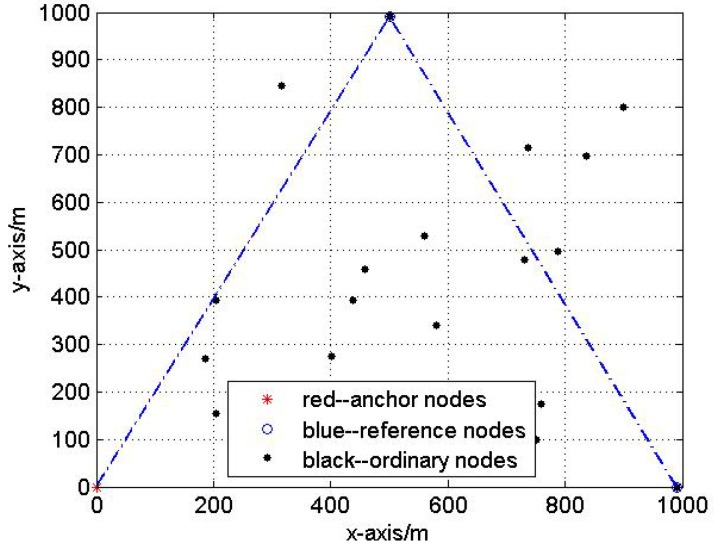
The optimal reference nodes selection.

**Figure 8:**
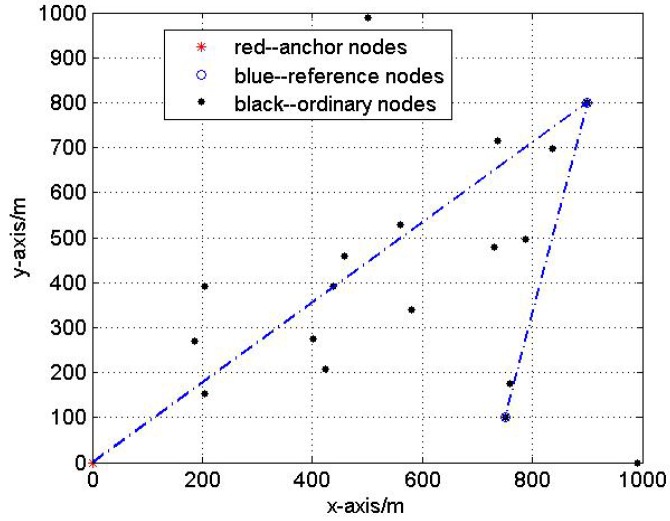
The non-optimal reference nodes selection.

**Figure 9:**
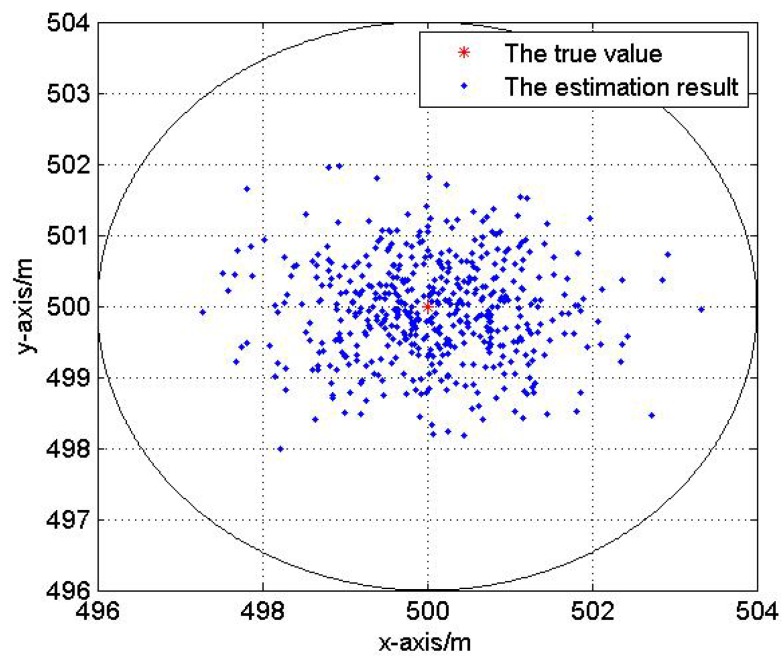
The localization result with optimal reference nodes.

**Figure 10:**
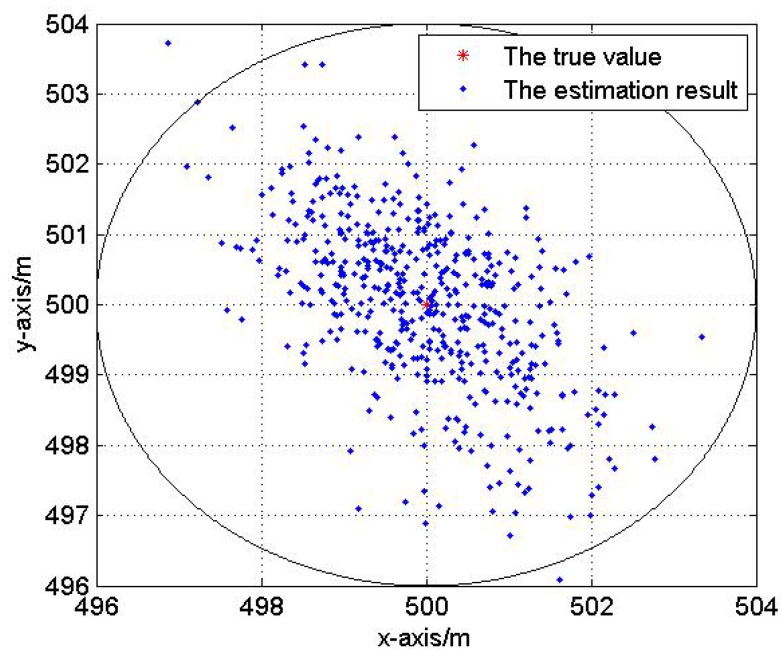
The localization result without optimal reference nodes.

In this test, we analyze the impact of reference nodes’ topology on the localization performance. Fix the Gaussian noise variance be 3 m. Five hundred Monte Carlo localization experiments are done with optimal and non-optimal reference nodes selections respectively in the same network. Figure 7 and Figure 8 show the optimal and non-optimal reference nodes selection results and 500 Monte Carlo experiments are exhibited in Figure 9 and Figure 10.

Figure 7, Figure 8, Figure 9 and Figure 10 demonstrate the effectiveness of the proposed reference nodes selection scheme. From Figure 9 and Figure 10 we can see that the optimal reference nodes can generate a better localization performance than non-optimal references.

#### 4.2.4. Test4: Communication Cost

In this test, we compare the communication cost during the localization procedure between the proposed DRL algorithm and other localization approaches.

Communication cost is the transmission data size during the localization procedure which is measured in bits. Nodes in UANs have limited power and communication cost is one of the important part of power consumption in each node. Therefore, communication cost is an essential index of the localization algorithm for UANs.

The sending data packet usually has two parts, a packet-header and packet-body. In the DRL algorithm, the packet-header involves the transmission modes, time stamps, node’s ID, etc., and the packet-body is the transmission data.

**Figure 11:**
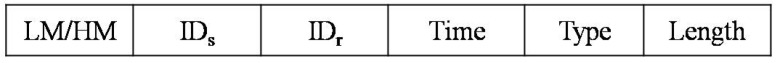
The packet-header.

We design the packet-header as in Figure 11.

M nodes are randomly deployed in the network.

LM/HM is the transmission mode, 1 bit;

ID${}_{s}$ is the ID of sending node, log${}_{2}M$ bits;

ID${}_{r}$ is the ID of receiving node, log${}_{2}M$ bits;

Time is the time stamp, 2 bytes;

Type is the type of sending data. There are two types of data in DRL, 2 bits;

(1) “Hello” packet: The “Hello” packet triggers the localization procedure and help nodes to measure the distance between each other.
(2) Range information packet: Range information packet includes all the distance information of nodes in the network.

Length is the length of the packet body in the sending data.

The length of the packet body is analyzed below.

As described above, there are two types of data in DRL and each of them has different length.

(1) “Hello” packet: Because “Hello” packet only triers the localization procedure and has no data to transmit, the packet length of “Hello” packet is 0;
(2) Range information packet: Range information packet includes all the distance information of any node between each other, thus the packet length is M byte;

(12) $$Communication\;Cost=\left(3M-2\right)log_{2}M+39M-20\;\left(bits\right)$$

**Figure 12:**
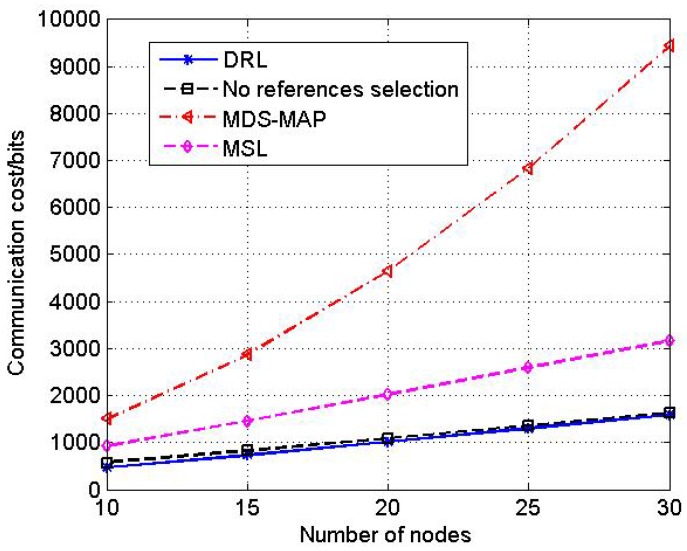
The comparison of communication cost.

Increase the number of nodes from 10 to 30. In Figure 12, we show the communication cost of different algorithms.

The result shows that the communication cost of all algorithms increases as the number of nodes grows. However, from Figure 12 we can see that the proposed DRL algorithm has a lower communication cost than the other three approaches, especially the MDS-MAP algorithm, which means that DRL can perform better in the low band-width underwater environment.

## 5. Experiment Results

**Figure 13:**
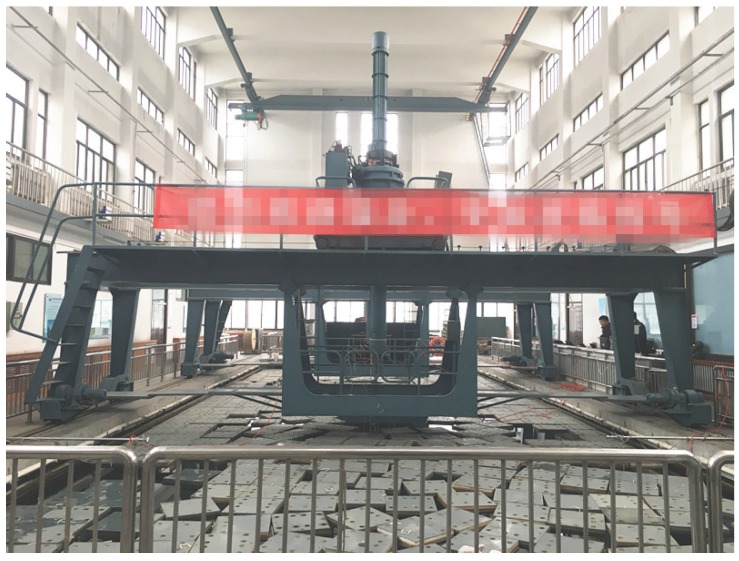
The anechoic tank laboratory.

**Figure 14:**
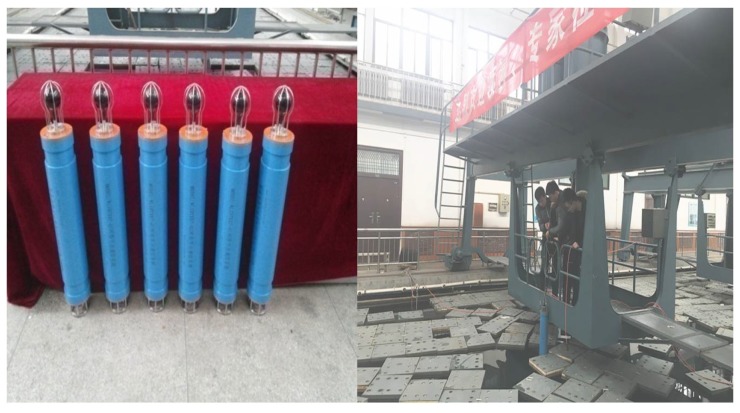
The experiment images.

To give a more concrete example, we tested the proposed DRL algorithm on the real experimental results which was done in the anechoic tank laboratory as shown in Figure 13 and Figure 14.

The experiment is limited by the scale of the laboratory. The designed UAN localization system contains five nodes and one of them is the anchor. Fix the anchor be (0,0) and change other nodes’ positions to get three different topologies. We test the localization performance on each topology. TDMA protocol and TOA ranging method are adopted in the designed network system.

**Figure 15:**
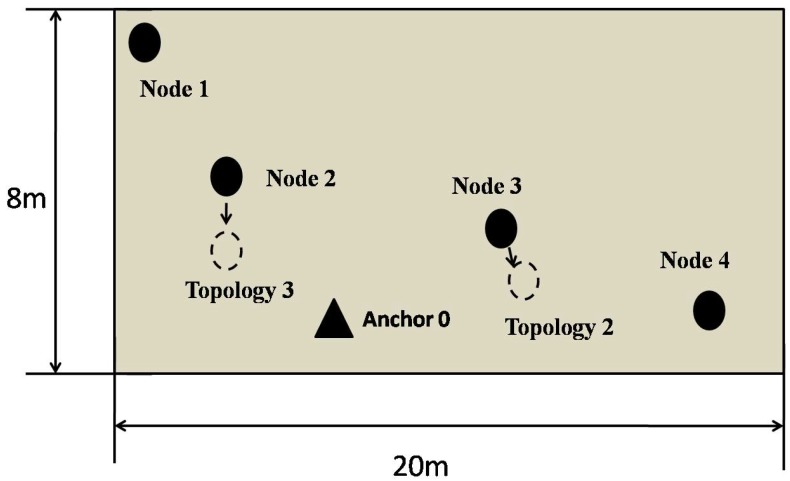
3 different topologies.

Figure 15 shows the structure of designed UAN system and the topology changes. In this experiment, the network was operated based on nodes’ IDs. The anchor is labeled as node 0, and so on. Five tests were done in each network topology.

(1) Topology 1: The solid line in Figure 15;
(2) Topology 2: Change node 3’s position to the site of dotted line;
(3) Topology 3: Change node 2’s position to the site of dotted line.

### 5.1. Ranging Experiment

We used the TOA ranging method to measure the distance of nodes between each other. The true value and one of the test results are shown in Table 1 and Table 2, respectively.

The ranging error of five tests are exhibited in Table 3. Based on the experimental results, the mean ranging error of the designed UAN system is about 0.0468 m.

### 5.2. Localization Experiment

On the basis of the ranging test, the localization experiments were done with three different topologies as described above. Five tests were done in each network topology as well.

#### 5.2.1. Topology 1

**Figure 16:**
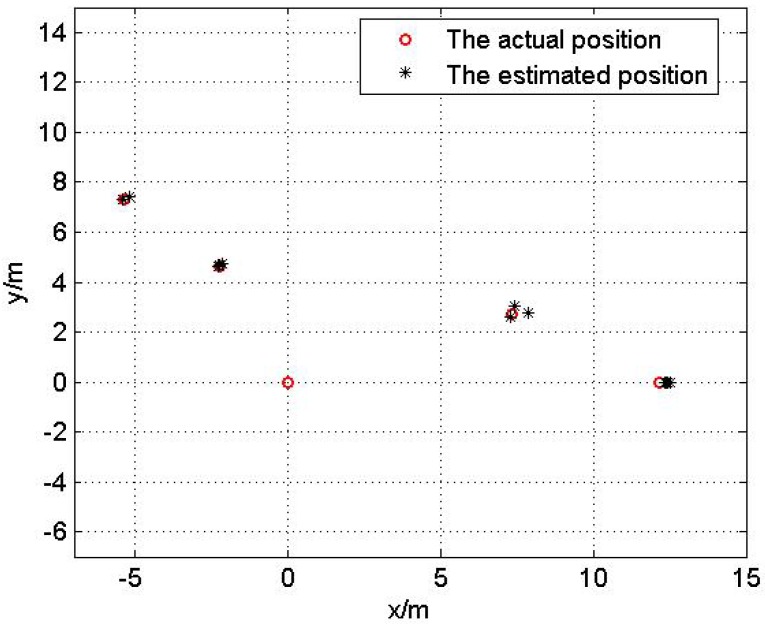
The localization result of topology 1.

The experimental result of topology 1 is shown in Figure 16 and the localization error is exhibited in Table 4.

#### 5.2.2. Topology 2

**Figure 17:**
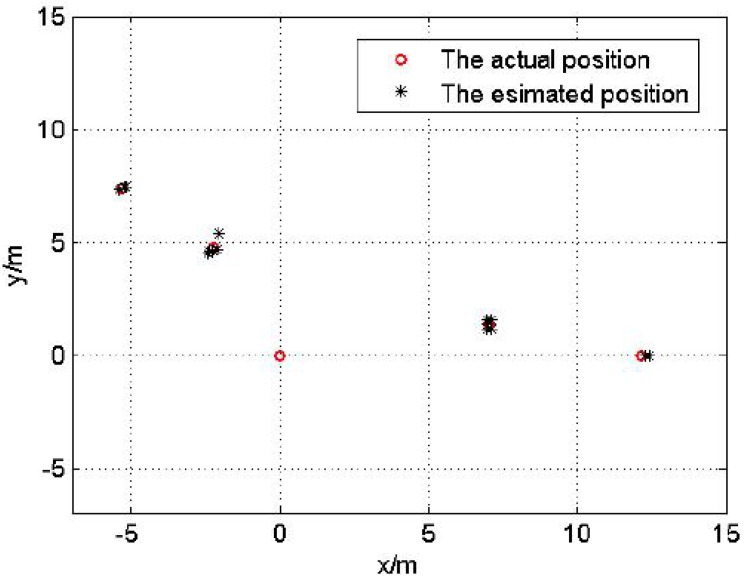
The localization result of topology 2.

The experiment result of topology 2 is shown in Figure 17 and the localization error is exhibited in Table 5.

#### 5.2.3. Topology 3

**Figure 18:**
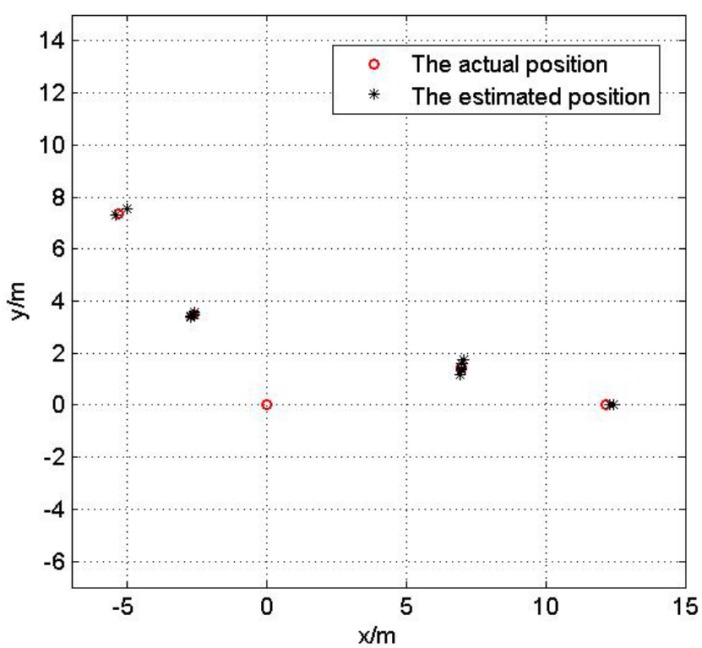
The localization result of topology 3.

The experimental result of topology 3 is shown in Figure 18 and the localization error is exhibited in Table 6.

According to the analysis of the experimental results we can see that the localization errors of the designed system in each topology are 0.1938 m, 0.2227 m, 0.1610 m which are related to the ranging accuracy, the network topology and the transducer response.

#### 5.2.4. The Localization Experiment of MDS-MAP

**Figure 19:**
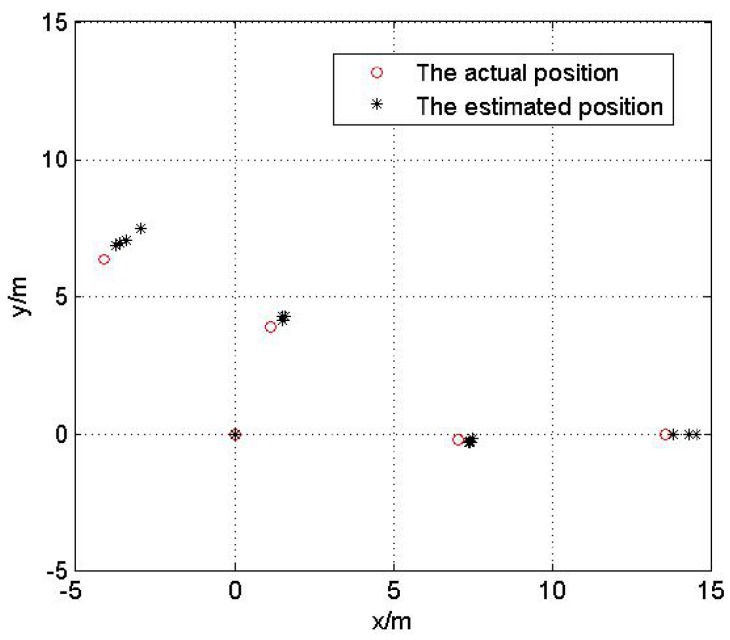
The localization result of MDS-MAP.

With the same network system, we test the MDS-MAP approach based on the real data. The experimental result and localization error are exhibited in Figure 19 and Table 7 respectively.

It is observed that, similar to the simulation results, the proposed DRL algorithm obtained a much smaller mean error than the classic MDS-MAP algorithm on the real test environment.

#### 5.2.5. Simulation Results

Simulation results of DRL and MDS-MAP algorithms with the same topology of experiment are as follows with the range error varying from 0.1 m to 0.4 m.

**Figure 20:**
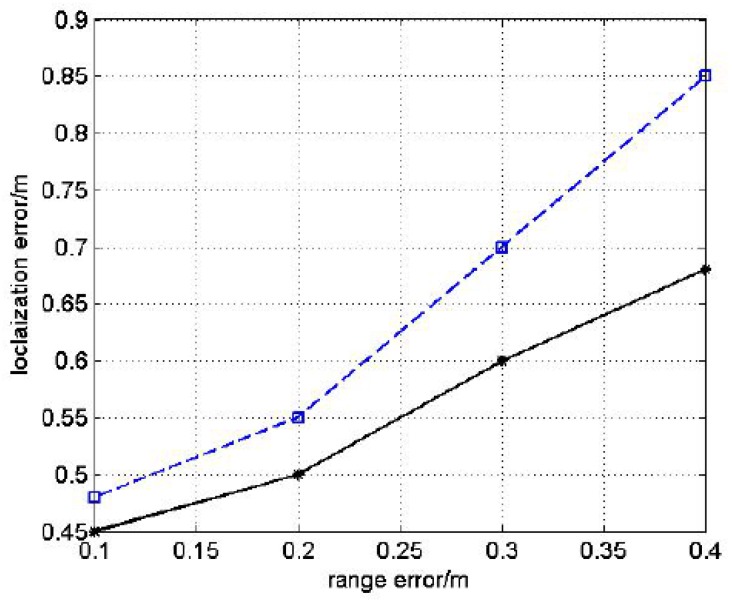
The simulation result.

From Figure 20, we can see that the simulation results are in accordance with the experiment results. Both of them demonstrate the effectiveness of the DRL algorithm.

## 6. Conclusions

In this paper, we proposed a double rate localization algorithm with one anchor for multi-hop underwater acoustic networks. The algorithm proposed a double rate scheme which separates the localization procedure into two modes, low-rate transmission mode and high-rate transmission mode, which can both increase the ranging measurement accuracy in multi-hop networks and maintain the transmission rate. Meanwhile, the algorithm also proposed an optimal references selection scheme used to find the best reference nodes in the algorithm that increase the localization accuracy for underwater networks with only one anchor. Simulated and experimental results demonstrated the performance advantage of the proposed DRL algorithm for underwater networks over the classic methods in various aspects. In the future, the localization algorithm for mobile UANs will be studied and we will improve the designed UANs system to accomplish more function.

## Figures and Tables

**Table 1:** The actual distance between each node/m.

ID	0	1	2	3	4
0	0	12.37	7.14	9.08	4.36
1	12.37	0	5.57	19.16	15.42
2	7.14	5.57	0	13.65	9.85
3	9.08	19.16	13.65	0	4.75
4	4.36	15.42	9.85	4.75	0

**Table 2:** The ranging measurement between each node/m.

ID	0	1	2	3	4
0	0	12.40	7.05	9.12	4.37
1	12.38	0	5.62	19.19	15.43
2	7.16	5.48	0	13.67	9.86
3	9.09	19.19	13.78	0	4.73
4	4.40	15.43	9.86	4.64	0

**Table 3:** The ranging error of 5 tests/m.

	Exp. 1	Exp. 2	Exp. 3	Exp. 4	Exp. 5	Mean Error
Error	0.0526	0.0541	0.0503	0.0377	0.0364	0.0468

**Table 4:** The localization results of 5 tests in topology 1/m.

	Exp. 1	Exp. 2	Exp. 3	Exp. 4	Exp. 5	Mean Error
Error	0.3599	0.1690	0.2040	0.1978	0.1826	0.2227

**Table 5:** The localization results of five tests in topology 2/m.

	Exp. 1	Exp. 2	Exp. 3	Exp. 4	Exp. 5	Mean Error
Error	0.1645	0.1550	0.2031	0.1968	0.1721	0.1783

**Table 6:** The localization results of 5 tests in topology 3/m.

	Exp. 1	Exp. 2	Exp. 3	Exp. 4	Exp. 5	Mean Error
Error	0.1999	0.2049	0.1454	0.1048	0.1500	0.1610

**Table 7:** The localization results of MDS-MAP/m.

	Exp. 1	Exp. 2	Exp. 3	Exp. 4	Exp. 5	Mean Error
Error	0.5397	0.5190	0.5306	0.5506	0.5172	0.5314
